# European Immunization Week 2020: European Vaccination Information Portal launched

**DOI:** 10.2807/1560-7917.ES.2020.25.16.2004232

**Published:** 2020-04-23

**Authors:** Eurosurveillance editorial team

**Affiliations:** 1 European Centre for Disease Prevention and Control (ECDC), Stockholm, Sweden

On the occasion of European Immunization Week (EIW) 2020, the European Vaccination Information Portal was launched on 21 April. This publically accessible website provides anyone interested in vaccination with accurate, objective and up-to-date evidence on vaccines and vaccination with an European Union (EU) perspective.

The project is an initiative of the EU, following the Council Recommendation on Strengthened Cooperation against Vaccine Preventable Diseases adopted in December 2018. The portal, which is available in all of the EU’s official languages, features an overview of the existing mechanisms in the EU to ensure that available vaccines conform to the highest standards of safety and effectiveness. It also highlights the benefits of vaccination to the individual and the community. 

The website was developed by the European Centre for Disease Prevention and Control (ECDC), in partnership with the European Commission (EC), specifically, the Directorate-General for Health and Food Safety (DG SANTE) and the European Medicines Agency (EMA).

EIW, which is led and coordinated by the World Health Organization Regional Office for Europe (WHO/Europe), is marked across the WHO European Region every April. It aims to raise awareness of the importance of immunisation for people’s health and well-being. ECDC supports the EIW campaign by providing scientific evidence on immunisation. Information on ECDC activities in the context of EIW can be found here.

